# Ribavirin for Crimean-Congo hemorrhagic fever: systematic review and meta-analysis

**DOI:** 10.1186/1471-2334-10-207

**Published:** 2010-07-13

**Authors:** Karla Soares-Weiser, Sherine Thomas, Gail Thomson G, Paul Garner

**Affiliations:** 1 Enhance Reviews Ltd, 5 Percy Street, Office 4, London, W1T 1DG, UK; 2 Tropical and Infectious Diseases Unit, Royal Liverpool University Hospital, UK; 3 Centre for Emergency Preparedness and Response, Health Protection Agency Porton, Salisbury, SP4 0JG, UK; 4 Cochrane Infectious Diseases Group, Liverpool School of Tropical Medicine, Pembroke Place, Liverpool L3 5QA, UK

## Abstract

**Background:**

Crimean-Congo hemorrhagic fever epidemics often occur in areas where health services are limited, and result in high case fatality rates. Besides intensive care, ribavirin is often recommended. A solid evidence base for the use of this drug will help justify assuring access to the drug in areas where epidemics are common.

**Methods:**

We carried out a systematic review of observational and experimental studies of people with suspected or confirmed Crimean-Congo hemorrhagic fever that included comparisons between patients given ribavirin and those not. We extracted data on mortality, hospital stay, and adverse events. Risk of bias was assessed using a standard checklist, and data were presented in meta-analytical graphs, stratified by study design, and GRADE tables presented. The risk of bias was summarised using the GRADE method.

**Results:**

21 unique studies, including one randomised controlled trial of ribavirin, were included. Quality of the evidence was very low, with a Down and Black median score of 4 (maximum possible 33). Ribavirin treatment was not shown to be superior to no ribavirin treatment for mortality rate in a single RCT (RR: 1.13, 95%CI: 0.29 to 4.32, 136 participants, GRADE=low quality evidence); but ribavirin was associated with reduced mortality by 44% when compared to no ribavirin treatment in the pooled observational studies (RR: 0.56, 95%CI: 0.35 to 0.90, 955 participants; GRADE=very low quality evidence). Adverse events were more common with the ribavirin patients, but no severe adverse events were reported. No difference in length of hospital stay was reported.

**Conclusions:**

No clear message of benefit is available from the current data on ribavirin as observational data are heavily confounded, and the one trial carried out has limited power. However, ribavirin could potentially have benefits in this condition and these results clearly indicate a pragmatic, randomised controlled trial in the context of good quality supportive care, is urgently needed and ethically justified.

## Background

Crimean-Congo hemorrhagic fever (CCHF) is a potentially fatal viral disease. The CCHF virus is a member of the Nairovirus genus of the Bunyaviridae family. This genus includes other species which are pathogens in humans such as the Dugbe virus and the Nairobi sheep disease virus [1,2]. It possesses 3 segments of negative-sense RNA [3,4] and an RNA dependant RNA polymerase packed within a lipid envelope which contains 2 viral glycoproteins [Gn and Gc]. This structure is characteristic of other members of the Bunyaviridae family.

The virus is transmitted to humans through tick bites or exposure to blood and tissues of infected animals. Different domestic and wild animals have been identified as a reservoir for this virus, including cattle, sheep, goats, hedgehogs and hares [5-8]. Numerous species of ticks can carry the virus, however very few of them have been implicated as vectors. The most important tick vector is the *Hyalomma spp*., as the virus was isolated from it and its geographic distribution coincides with that of the disease [9]. Another transmission route of the virus in humans is through contact with the blood of an infected person during the acute phase of the disease [10]. This is especially significant among healthcare workers who may be infected while treating CCHF patients during an outbreak [11].

One of the most important features of the virus is its diverse geographic distribution including Africa, Asia, Eastern Europe and the Middle East [12], making it the most widespread tick-borne virus infecting humans. Outbreaks have been documented in all these areas since the 1960 s, with the most recent cases coming from Iran [13] and Turkey [14]. In addition, climatic, environmental and agricultural changes may affect the distribution of the tick vector and influence the location and timing of outbreaks.

The pathogenesis of CCHF remains elusive, mainly due to lack of adequate animal models and laboratories with the proper bio-safety containment level. Studies in human patients reveal endothelial damage resulting from either direct infection of the cells or indirect effect of viral and host factors [15,16]. The clinical features of CCHF are divided into four periods - incubation, pre-hemorrhagic, hemorrhagic, and convalescence [9]. The incubation period may vary between 2-9 days according to the transmission route [10]. This may be followed by a sudden onset of signs such as fever, headache, myalgia, arthralgia, abdominal pain and vomiting. Additional signs may also appear including sore throat, conjunctivitis, jaundice, photophobia and various sensory and mood alterations. In severe cases, hemorrhagic manifestations may appear as early as 3-6 days following disease onset. Petechiae and ecchymosis of the skin and mucous membranes, as well as gastrointestinal bleeding are the most common signs at this stage, while cerebral hemorrhage and liver necrosis reveal a more severe manifestation with poorer prognosis [14]. Mortality rates usually range between 5-50% [9], although numbers as high as 80% have been reported sporadically [6].

Early diagnosis is essential in CCHF cases and is currently possible using first line molecular methods for rapid diagnosis such as reverse transcription PCR (RT-PCR) and real-time PCR. Serological methods such as ELISA and immunofluorescent assays may also provide a sensitive and specific diagnosis approximately 7 days following disease onset. Different cell line cultures and inoculation of the virus into mice may be used for virus isolation [14].

Prompt supportive treatment including blood products administration is the major current therapeutic option, although several attempts have been made in the past to treat patients with immunoglobulins produced from vaccinated horses [9] and with serum taken from convalescing CCHF patients [17]. To date, however, no clinical trials have been reported testing the latter interventions. More recently, the antiviral drug ribavirin - a synthetic purine nucleoside analogue synthesized in 1972 [18] - has revealed promising activity against the CCHF virus in vitro [19] and in an animal model of mice [20]. Several observational studies suggest efficacy of ribavirin in human patients [21-23], while evidence from randomised controlled clinical studies is lacking.

As CCHF activity appears to be increasing, particularly in European regions [24,25], it becomes essential to assess the effectiveness of ribavirin treatment. If results indicate that treatment with ribavirin is promising, efforts will need to be made to ensure its availability in areas of the world where CCHF is present. On the other hand, if the evidence is of poor quality and results remain inconclusive, focus will need to be targeted on securing better data as well as diminishing harm experienced by the patient. At the moment, there appears to be a gap between strongly held clinical beliefs and actual provision of ribavirin.

Our goal was to appraise and summarise the evidence about benefits and harms of ribavirin for treating CCHF in humans. Secondary objectives were to evaluate the effects of ribavirin, according to severity of disease and number of days from onset of illness that the drug was started, duration of ribavirin treatment, via of administration; and to evaluate whether prophylactic use following exposure to CCHF virus should be recommended.

## Methods

### Inclusion criteria

#### Type of studies

Randomised trials (RCTs) or observational studies comparing the efficacy of ribavirin with any other intervention or no treatment. Case series were limited to the ones with more than 10 cases, and were only summarised qualitatively.

#### Types of participants

Children and adults of any age, with a suspected or confirmed diagnosis of CCHF.

#### Types of interventions

Ribavirin compared to any other intervention or no treatment, regardless of via of administration or schedule.

#### Outcome measures

The primary outcome was the rate of mortality among intervention and control groups. Secondary outcomes were: (i) rate of mortality among those receiving ribavirin treatment in the first 5 days from the onset of symptoms or later than 6 days; (ii) duration of hospital stay; (iii) improvement of disease symptoms; (iv) time to recovery from symptoms; (v) serious adverse events that could be fatal or lead to treatment discontinuation; (vi) any reported adverse event; and (vii) the development of CCHF after prophylactic use of ribavirin in health care workers.

If information was available, all outcomes were categorised according to severity of disease, number of days from possible exposure to onset of symptoms, duration of treatment, severity of gastrointestinal symptoms, and via of administration.

### Search strategy

We attempted to identify all relevant studies regardless of language or publication status (published, unpublished). Search strategies were developed from insertion until September 2009 for the following databases: MEDLINE http://www.nlm.nih.gov/databases/databases_medline.html, EMBASE http://www.embase.com/, The Cochrane Library (2009, Issue 3), Current Controlled Trials Register, and ISI Citation Indexes at Web of Science (ISI). In addition, the internet was searched via general search engines, such as Google Scholar, for relevant studies and the reference lists of the included studies were also checked. Details of search strategies can be found in the online material (Additional file 1. Search Strategy).

### Data collection and assessment

This systematic review followed the Cochrane Collaboration Handbook [26].

Two reviewers independently inspected the abstract of each reference identified by the search. For potentially relevant articles, or in cases of disagreement, the full article was obtained, independently inspected and inclusion criteria were applied. Any disagreement was resolved through discussion and the abstract was checked by a third reviewer. Justifications for excluding studies from the review were documented.

Data extraction forms were developed and piloted independently on a small selection of studies varying in quality. Two reviewers independently extracted information on study population, setting, details of intervention(s) used and outcomes. Data extraction was discussed and decisions documented. Studies were identified using the name of the first author and year in which the study was first published. Risk of bias was assessed independently by two reviewers using the Downs & Black checklist for observational studies [27] and summarized using the GRADE methodology [28]. Any disagreements were resolved by consensus.

### Statistical analyses

Statistical analyses were performed using Comprehensive Meta-analysis (version 2). Dichotomous data was analysed by calculating the risk ratio (RR) for each study and the correspondent 95% confidence intervals. Continuous data was analysed by calculating the standardised difference in means (SMD) for each study and the correspondent 95% confidence intervals [26]. All analyses were conducted using the random-effects model (inverse of variance method), as we combined studies with different methodological design and assumed that the true effect size varied from study to study [29].

Presence of statistical heterogeneity was assessed with the Q-test (considered significant for p < 0.10) [30] and quantified using I-squared (I^2^) [31,32]. In addition, the between-study variance (random effects) was calculated by estimating the standard deviation of underlying effects across studies (TAU) [26].

We planned to conduct subgroup analyses to investigate whether the effects of ribavirin differs according to: CCHF severity of symptoms, number of days from onset of symptoms to starting the drug, duration of treatment, severity of gastrointestinal symptoms and via of administration. Only data on duration of treatment was provided in three studies and analysed separately.

## Results

### Search results

**Figure 1:**
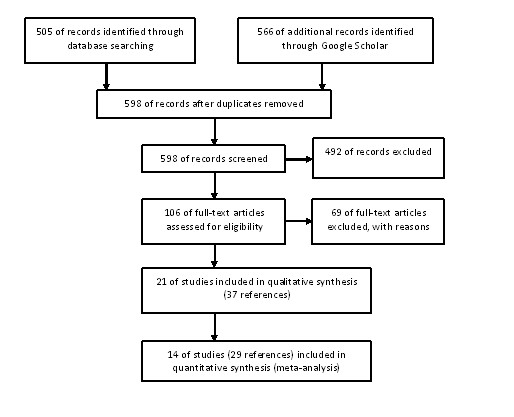
**Flow chart of studies included in this review**.

We retrieved 1071 references from database and internet site searches. After manually removing duplicates, two reviewers independently screened 598 articles, and the full report of 106 potentially relevant studies was obtained. Of these, we included 21 studies (37 references) and excluded 69 references (Figure 1). The primary reasons for excluding studies were as follows: case series with less than 10 cases reported (n = 20), ribavirin was not used to treat CCHF (n = 24), not original studies (n = 13), not about CCHF (n = 8) and other reasons (n = 4).

Ultimately, two randomised trials [33,34], nine historical control [22,35-42], one case-control [43], two cohort [44,45], two cross-sectional studies [46,47] and five case-series with more than 10 participants [48-52] were included in the current review. Studies were conducted in Iran [22,34,35,46,47,49], Pakistan [42,50,52], Turkey [33,36-41,43-45,48] and Russia [51].

### Study characteristics

#### Participants

In one study, participants were children and adolescents with an average age of 13.3 (SD = 4.6) years [46]; all participants in the other studies were adults and the average age ranged from 26 to 54 years old. The percentage of females in the studies ranged from 22% to 68%. Eight studies included only participants with a confirmed diagnosed of CCHF (confirmed cases by using PCR, ELISA and/or viral culture), five included suspected or confirmed cases, but reported outcomes only for those on which the diagnosis of CCHF was confirmed and seven studies included participants with a clinical or confirmed diagnosis of CCHF. The sample size of the included studies varied from 8 to 283 participants. Few studies reported the proportion of participants with co-morbidities (Additional file 2. Description of studies). A diagnosis of CCHF was confirmed by IgM and IgG antibodies in 18 studies and no information was provided in two studies; detection of viral antigens were used in 12 studies. Additional viral culture was used in two studies to confirm CCHF diagnosis (Additional file 2. Description of studies).

#### Interventions

Except for one study, where ribavirin was administered intravenously [37], oral ribavirin was given within a median of three to seven days after onset of symptoms. The dosage recommended by the World Health Organization (WHO) (30 mg/kg as an initial loading dose, then 15 mg/kg every 6 h for 4 days, and then 7.5 mg/kg every 8 h for 6 days) was used in 10 studies, different dosage was used in three studies and seven studies did not report the dosage of ribavirin used. Fourteen studies reported the use of supportive therapy and blood products and this information was not provided in six studies (Additional file 2. Description of studies).

#### Outcomes

The primary outcome, mortality, was reported in 12 studies comparing ribavirin vs. no ribavirin treatment, in three studies comparing ribavirin treatment initiated in the first 5 days of onset or later, and in one study comparing ribavirin treatment with ribavirin and immunoglobulin. Adverse events (not described as serious or leading to treatment discontinuation) were reported in four studies and length of hospitalisation was reported in four studies. All other outcomes were not reported.

### Methodological assessment

**Figure 2:**
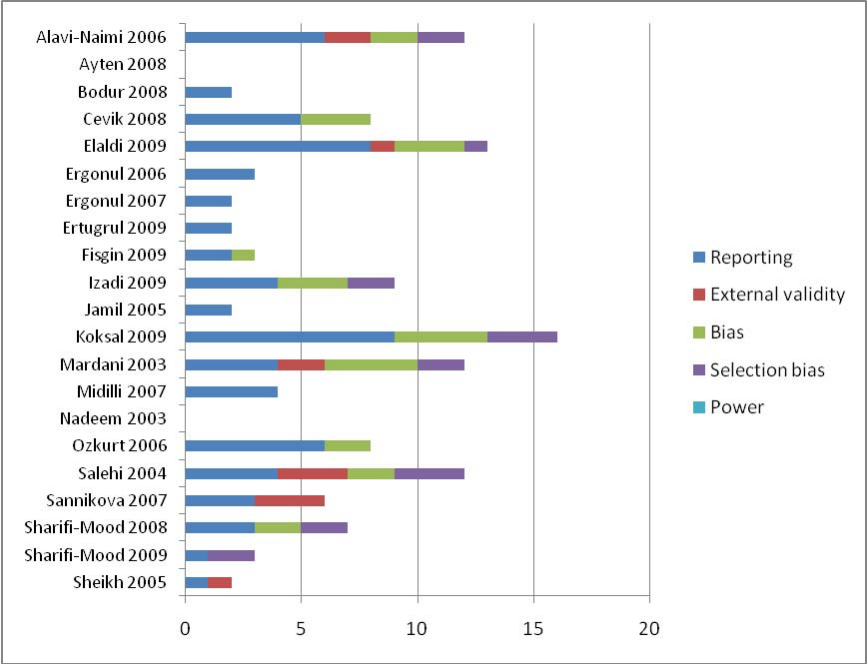
**Risk of bias assessment based on Downs & Black Checklist**.

Figure 2 summarises methodological characteristics of all included studies. Further details are provided in the online material (Additional file 3. Risk of bias of included studies). Downs & Black [27] final score on the quality of the 21 included studies ranged from 0 to 16 with a median score of 4 (maximum possible score was 33). Scores for each of the five factors devised by Downs & Black [27] varied from 0 to 9 for quality of reporting (maximum score = 11), 0 to 3 for external validity (maximum score = 3), 0 to 4 for internal validity bias (maximum score = 8), 0 to 3 for internal validity confounding (maximum score = 6) and all studies received a 0 for power calculation (maximum score = 5).

### Effects of interventions

#### Mortality rate: quantitative synthesis

**Figure 3:**
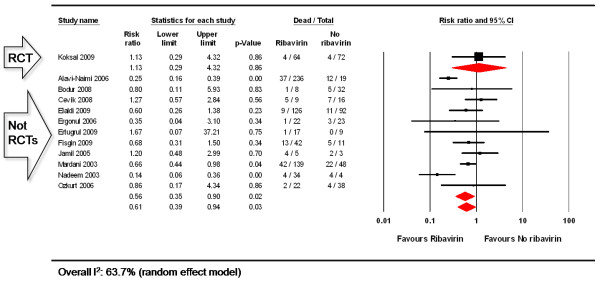
**Rate of mortality in patients with CCHF: ribavirin vs. no ribavirin treatment**.

One RCT [33] and 11 observational studies [22,35-43,50] provided data on rate of mortality comparing ribavirin vs. no ribavirin treatment for CCHF (Figure 3). Ribavirin treatment was not superior to no ribavirin treatment to reduce the mortality rate in a single RCT (RR: 1.13, 95%CI: 0.29, 4.32, 136 participants). Ribavirin reduced mortality by 44% when compared to no ribavirin treatment in the pooled observational studies (RR: 0.56, 95%CI: 0.35, 0.90, 955 participants). Significant heterogeneity was observed for the pooled observational studies (Q = 29.02, p = 0.001, I^2 ^= 65%).

#### Mortality rate: qualitative synthesis

One study, published in Persian, reported results of a randomised, single-blinded controlled study on which oral ribavirin with intravenous immunoglobulin was compared to oral ribavirin in only 60 CCHF patients [33]. Data is provided only for the 40 confirmed CCHF cases. Twelve patients received ribavirin plus immunoglobulin (intervention group) and 28 patients received only ribavirin (control group) during one week; patients were followed up for 8 weeks. Authors reported three deaths in each group.

In an additional five studies [45,46,48,51,52] all participants received ribavirin treatment and rates of mortality were provided:

Ayten (2008) [48] described 63 adults (mean age: 46, SD: 16.9 years) from Eastern Turkey with a confirmed case of CCHF. Oral ribavirin was prescribed to 46 (73%) and a case-fatality ratio of 4.8% was reported, although it is unclear if this was only among those receiving ribavirin.

Midilli (2007) [45] investigated the genetic diversity of the virus in 91 suspected cases of CCHF. In ten patients CCHF was confirmed using semi-nested polymerase chain reaction (PCR) following RT-PCR. Oral ribavirin was prescribed to all 10 confirmed cases (dose not reported) and no deaths were reported.

Sannikova (2007) [51] described a series of 283 adults (age not reported) from Russia with CCHF. Oral ribavirin was prescribed to all of them at a dose of 1000 to 1200 g per day for two days and no deaths were reported.

Sharifi-Mood (2008) [46] described clinical and epidemiologic features of CCHF among 34 children and adolescents (mean age: 13.3, SD: 4.6 years) from a highly endemic region in Iran. Clinical manifestations were described as being similar to those in adults. The study reported a case-fatality ratio of 26.5% (9 of 34).

Sheikh (2005) [52] described 135 suspected cases of CCHF, based on a five-year hospital based case series in the province of Balochistan in Pakistan. Eighty-three confirmed cases of CCHF were treated with oral ribavirin and the study reported a case-fatality ratio was 9.6% (8 of 83).

### Adverse events

One RCT [33] and three observational studies [37,39,43] provided data on adverse events comparing ribavirin vs. no ribavirin treatment to treat CCHF (Figure A of the online material). None of the adverse events were described as serious, or needed discontinuation of treatment and one of the studies reported no adverse events [40]. In the other three studies the observed adverse events were as follows. In Koksal 2009 [33] two patients developed serious anaemia and fatigue and these patients had to be hospitalised for a longer period of time. Cevik 2008 [37] was the only included study evaluating intravenous ribavirin in patients with severe CCHF; one patient developed an allergic maculopapular rash that was treated with antihistamines and two patients had nausea and vomiting due to intravenous ribavirin, and received symptomatic treatment. Ozkurt 2006 [43] described one case of mild hemolytic anemia due to ribavirin that recovered spontaneously after two days without withdrawing the drug. Ribavirin treatment did not cause more adverse events than no ribavirin treatment in a single RCT (RR: 5.62, 95%CI: 0.27, 114.83, 136 participants), or in the pooled observational studies (RR: 8.12, 95%CI: 0.97, 67.59, 85 participants, I^2 ^= 0%).

### Length of hospital stay

**Figure 4:**
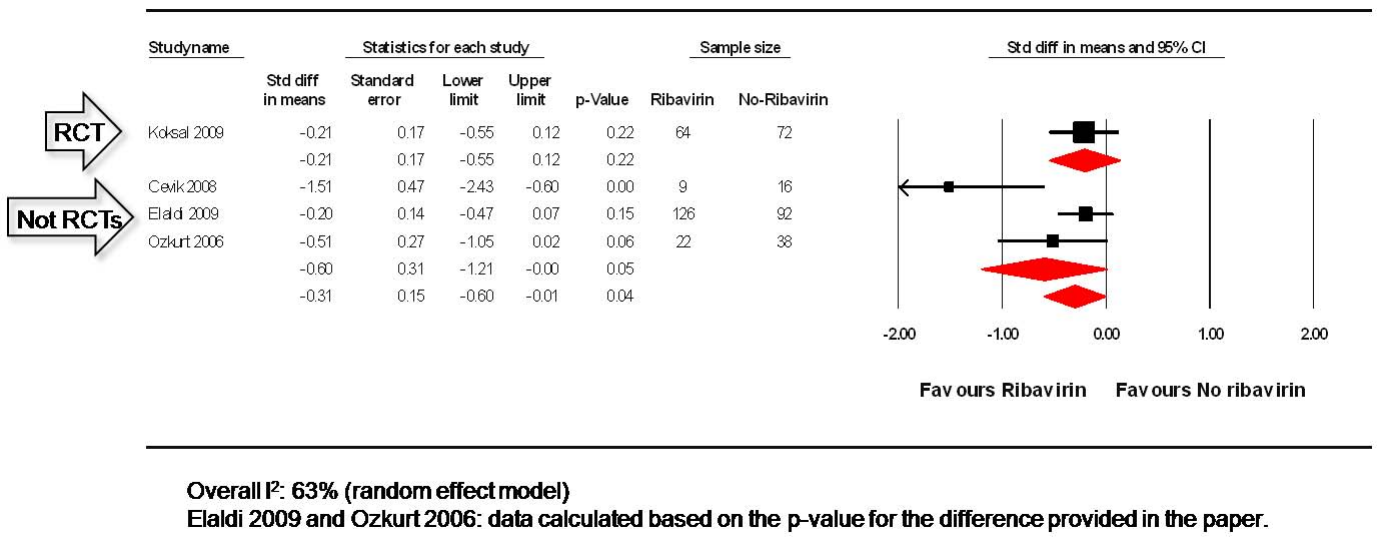
**Length of hospital stay in patients with CCHF: ribavirin vs. no ribavirin treatment**.

One RCT [33] and one observational study [36] provided information on the average length of hospital stay comparing patients using ribavirin vs. no ribavirin treatment to treat CCHF. Two other studies provided the p-value for the difference instead of the average length of hospital stay in days [38,43]. In order to merge the latter studies with the other two, we converted the data into the same effect size assuming a two-tailed p-value and independent, unmatched groups [29]. Pooled analysis can be seen in Figure 4. No difference was observed when those receiving ribavirin were compared to no ribavirin treatment in a single RCT (SMD: -0.21, 95%CI: -0.55, 0.12, 136 participants), or in the pooled observational studies (SMD: -0.60, 95%CI: -1.21, 0.00, 308 participants. Significant heterogeneity was observed for the pooled observational studies (Q = 7.74, p = 0.021, I^2 ^= 74%).

### Antibody responses following exposure to CCHF virus

One included study [44] aimed to detect antibodies against CCHF virus in healthcare workers in one of the largest referral tertiary-care community hospitals in Turkey. The sera from 75 healthcare workers were collected one month after the last admitted hospital case (October 2003), and tested for CCHF IgM and IgG by ELISA. Eighty three percent of the healthcare workers were at risk of exposure to the body fluids of patients, but only one was CCHF IgG positive. Authors reported that the high rate of compliance to the universal precautions protected healthcare workers against CCHF infection. No one in this study was given prophylactic ribavirin.

### Sub-group analysis

Three observational studies [41,47,49] provided data comparing ribavirin treatment starting before or after 5 days of onset of disease. No statistically significant difference was observed between groups (RR: 0.31, 95%CI: 0.08 to 1.25, 344 participants). Significant heterogeneity was observed for the pooled observational studies (Q = 13.52, p = 0.001, I^2 ^= 85%).

### GRADE assessment

Table 1 provides an assessment of the effect estimates and the robustness of the data using the GRADE methodology. Ribavirin effects from observational data are of very low quality.

**Table 1:** Summary of findings: ribavirin vs. no ribavirin for treating patients with CCHF (population: patients with CCHF; settings: hospital based; intervention: ribavirin)

Outcomes	Illustrative comparative risks* (95% CI)		Relative effect (95% CI)	No of Participants (studies)	Quality of Comments the evidence (GRADE)
	Assumed risk	Corresponding risk			
	Control	ribavirin			
**Mortality (RCT)** Follow-up: mean 8 weeks	**56 per 1000**	**63 per 1000** (16 to 242)	**RR 1.13** (0.29 to 4.32)	136 (1 study)	⊕⊕⊖⊖ **low**^1,2^

**Mortality (Observational studies)** Follow-up: 1-12 months	**254 per 1000**	**142 per 1000** (89 to 229)	**RR 0.56** (0.35 to 0.9)	955 (11 studies)	⊕⊖⊖⊖ **Very low**^3,4,5,6^

**Length of Hospital stay (RCT)** Days in hospital Follow-up: mean 8 weeks	The mean length of hospital stay (rct) in the control groups was **6.3 days**	The mean Length of hospital stay (RCT) in the intervention groups was **0.21 standard deviations lower **(0.55 lower to 0.12 higher)		136 (1 study)	⊕⊖⊖⊖ **very low**^1,2^

**Length of Hospital Stay (Observational studies)** days in hospital Follow-up: mean 4-24 months	The mean length of hospital stay (observational studies) in the control groups was **6.4 days**	The mean Length of hospital stay (Observational studies) in the intervention groups was **0.60 standard deviations lower **(1.21 lower to 0 higher)		303 (3 studies)	⊕⊖⊖⊖ **Very low**^3,5,6,7^

*The basis for the **assumed risk **(e.g. the median control group risk across studies) is provided in footnotes. The **corresponding risk **(and its 95% confidence interval) is based on the assumed risk in the comparison group and the **relative effect **of the intervention (and its 95% CI). **CI: **Confidence interval; **RR: **Risk ratio

GRADE Working Group grades of evidence

**High quality: **Further research is very unlikely to change our confidence in the estimate of effect.

**Moderate quality: **Further research is likely to have an important impact on our confidence in the estimate of effect and may change the estimate.

**Low quality: **Further research is very likely to have an important impact on our confidence in the estimate of effect and is likely to change the estimate.

**Very low quality: **We are very uncertain about the estimate.

Assessment notes

^1 ^No details of allocation concealment and method of randomisation was provided. Open label.

^2 ^Wide confidence intervals.

^3 ^Details of study design and population characteristics not provided in most studies.

^4 ^Two studies (Alavi-Naimi 2006 and Nadeen 2003) were significantly more favourable to ribavirin than the other 9 included observational studies.

^5 ^Although there was no significant lateral asymmetry on the funnel plot, and Egger's regression was not significant (intercept: 0.829, 2-tailed p-value = 0.408), we cannot completely discard publication bias because all studies were observational and published onl in Iran, Pakistan and Turkey.

^6 ^Most studies used historical controls and provided little or no information about how patients were selected, whether they represent the whole population in risk or characteristics of included patients.

^7 ^One study (Cevik 2008) was significantly more favourable to ribavirin than the other 2 included observational studies.

## Discussion and Conclusions

Evidence that ribavirin has an effect on outcome in patients is limited. Trial data are confined to one recently conducted randomized, placebo-controlled trial. The confidence intervals are very wide, and so it is not possible to be confident about any effect from this data. Observational data suggest benefit, across the 11 studies, with confidence intervals that are significant. The point estimate suggests a potentially large effect, but the quality of the data is very low (see table 1), and the quality assessment shows a high risk of bias. Indeed, severity of illness is likely to confound the relationship: less severely ill patients survived long enough after admission to receive ribavirin, whereas the sicker patients may die sooner, and thus be less likely to receive ribavirin. Whilst studies are all consistent with benefit, we must guard against spurious precision from confounded association [53].

Also factors including include platelet count, white blood cell counts, INR and gastrointestinal hemorrhage have previously been shown to influence the outcome of CCHF cases, and these have previously been described in prognostic scoring systems [39]. Unfortunately, the data from many of the trials reviewed here was limited and severity scores were not used uniformly, therefore this was difficult to assess. Severity criteria should also be taken into account in any future trials looking at ribavirin use in CCHF.

Adverse effects are more common with ribavirin than with no treatment, but none were described as serious or required discontinuation of treatment. However, the number of studies and patients in whom this was reported was low.

The mortality varies considerably between the case series and the trial. This may be a result of case mix (with some of the older case series detecting seriously ill people), supportive management that has been improving over time, or simply the trial effect, with falls in mortality in both treatment and control arms, as the trial environment generally improves care.

### Limitations of the current evidence

Carrying out a randomised trial in this severe infectious disease is commendable and the trial reported follows good scientific principles. However, the strength of this review would have been greatly enhanced by a more complete reporting of data in the original papers. In particular, the single RCT did not report a procedure for randomisation, a method to guarantee an adequate allocation concealment and whether patients and/or healthcare workers were blinded to group assignment [33].

Furthermore, the final Downs & Black [27] quality score for the observational studies ranged from 0 to 16 with a median score of 4 (maximum possible score was 33), showing that even if carried out adequately, the study design has not been consistently reported.

### Applicability

The patient population in the included trials was usually young adults, with one study reporting CCHF in children. Information concerning the risk factors of these patients and the mechanism of exposure to CCHF virus is scarce, as patient characteristics were rarely detailed. In addition, all included studies contributing data for this review were conducted in three countries (Iran, Pakistan, and Turkey), with many other countries reported as endemic areas [54] failing to report results of CCHF treatment or whether treatment with ribavirin is a current practice in these areas.

The World Health Organization has approved ribavirin for use for CCHF and has added the drug to the essential drug list, mainly based on its in vitro effect (reference WHO Essential drugs submission) [55]. The drug is not cheap, therefore if indeed it is effective, it is important that its distribution is assured, that it is available immediately when an outbreak occurs, and health staff have clear procedures about when and how to use the drug. This will require substantive investment in purchase, distribution, and training in areas where health services are often fairly basic. A clear message from research evidence of the benefit of giving this drug, and the size of the effect, will be of considerable help in justifying the investment required in ensuring all patients receive this drug promptly, and hence the need for further trials.

### Implications for practice

Current research evidence is insufficient to be sure ribavirin is of benefit in this condition. Observational data are compatible with an effect, but are confounded and it is not possible to rely on this data alone. The estimates from randomised comparisons are compatible with both no effect and a substantive benefit.

### Implications for future research

There is an urgent need to rapidly establish a multi-centred, simple mortality trial of ribavirin in CCHF.

## Abbreviations

CCHF: (Crimean-Congo Hemorrhagic Fever); ELISA: (Enzyme-linked immunosorbent assay); EMBASE: (Excerpta Medica Database); GRADE: (The Grading of Recommendations Assessment, Development and evaluation approach); I^2 ^(I-squared statistic); MEDLINE: (Medical Literature Analysis and Retrieval System Online); RCT: (randomized-controlled trial); RT-PCR: (Real-time polymerase chain reaction; RR: (Risk Ratio); SMD: (Standardised difference in means); TAU: (Standard deviation of underlying effects across studies); WHO: (World Health Organization).

## Pre-publication history

The pre-publication history for this paper can be accessed here:

http://www.biomedcentral.com/1471-2334/10/207/prepub

## Supplementary Material

Additional file 1 **Search strategy**. Click here for file

Additional file 2 **Description of studies (table)**. Click here for file

Additional file 3 **Risk of bias of included studies (table)**. Click here for file
