# Supplementary material for: Ribavirin for Crimean-Congo hemorrhagic fever: systematic review and meta-analysis
Source: BMC Infect Dis. 2010 Jul 13;10:207. doi: 10.1186/1471-2334-10-207 (PMC2912908; doi:10.1186/1471-2334-10-207)
Supplement: Additional file 1 — Search strategy. [file 1471-2334-10-207-S1.DOCX]

Title: Search strategy

Description: This outlines the search strategy we used and the results for identifying studies

Medline: Search strategy performed on PUBMED (13^th^ September 2009)

| **#** | **Search strategy** | **Result** |
| --- | --- | --- |
| [#9](http://www.ncbi.nlm.nih.gov/sites/?querykey=21&dbase=pubmed&tab=History&querytype=eSearch&) | Search **#5 and #8** | [150](http://www.ncbi.nlm.nih.gov/sites/?cmd=HistorySearch&querykey=21&tab=&) |
| [#8](http://www.ncbi.nlm.nih.gov/sites/?querykey=20&dbase=pubmed&tab=History&querytype=eSearch&) | Search **#6 or #7** | [7282](http://www.ncbi.nlm.nih.gov/sites/?cmd=HistorySearch&querykey=20&tab=&) |

| [#7](http://www.ncbi.nlm.nih.gov/sites/?querykey=19&dbase=pubmed&tab=History&querytype=eSearch&) | Search **tribavirin or virazole or ribavirin** | [7282](http://www.ncbi.nlm.nih.gov/sites/?cmd=HistorySearch&querykey=19&tab=&) |
| --- | --- | --- |
| [#6](http://www.ncbi.nlm.nih.gov/sites/?querykey=18&dbase=pubmed&tab=History&querytype=eSearch&) | Search **("Ribavirin"[Mesh] OR "ribavirin 5'-diphosphate "[Substance Name] OR "ribavirin-5'-phosphate "[Substance Name] OR "5'-O-galactopyranosyl ribavirin "[Substance Name] OR "5'-O-glucopyranosyl ribavirin "[Substance Name] OR "ribavirin 5'-triphosphate "[Substance Name] OR "5'-nor carbocyclic ribavirin "[Substance Name] OR "ribavirin 3',5'-phosphate pentadecamer homoribopolymer "[Substance Name] OR "ribavirin 5'-sulfamate "[Substance Name] OR "tributylribavirin "[Substance Name] OR "ribavirin 2',3',5'-triacetate "[Substance Name] OR "ribavirin amidine "[Substance Name])** | [5498](http://www.ncbi.nlm.nih.gov/sites/?cmd=HistorySearch&querykey=18&tab=&) |
| [#5](http://www.ncbi.nlm.nih.gov/sites/?querykey=15&dbase=pubmed&tab=History&querytype=eSearch&) | Search **#1 or #2 or #3 or #4** | [10022](http://www.ncbi.nlm.nih.gov/sites/?cmd=HistorySearch&querykey=15&tab=&) |
| [#4](http://www.ncbi.nlm.nih.gov/sites/?querykey=14&dbase=pubmed&tab=History&querytype=eSearch&) | Search **((Crimea* or Congo) and (hemorrhag*))** | [815](http://www.ncbi.nlm.nih.gov/sites/?cmd=HistorySearch&querykey=14&tab=&) |
| [#3](http://www.ncbi.nlm.nih.gov/sites/?querykey=13&dbase=pubmed&tab=History&querytype=eSearch&) | Search **((Crimea* or Congo) and (fever))** | [925](http://www.ncbi.nlm.nih.gov/sites/?cmd=HistorySearch&querykey=13&tab=&) |
| [#2](http://www.ncbi.nlm.nih.gov/sites/?querykey=10&dbase=pubmed&tab=History&querytype=eSearch&) | Search **"Hemorrhagic Fever Virus, Crimean-Congo"[Mesh]** | [301](http://www.ncbi.nlm.nih.gov/sites/?cmd=HistorySearch&querykey=10&tab=&) |
| [#1](http://www.ncbi.nlm.nih.gov/sites/?querykey=8&dbase=pubmed&tab=History&querytype=eSearch&) | Search **"Hemorrhagic Fever, Crimean"[Mesh] OR "Hemorrhagic Fevers, Viral"[Mesh]** | [9560](http://www.ncbi.nlm.nih.gov/sites/?cmd=HistorySearch&querykey=8&tab=&) |

Embase ([www.embase.com](http://www.embase.com)): Search strategy performed on 25^th^ September 2009

| **#** | **Search strategy** | **Result** |
| --- | --- | --- |
| [#15](http://www.ncbi.nlm.nih.gov/sites/?querykey=19&dbase=pubmed&tab=History&querytype=eSearch&) | #6 AND #14 | [249](http://www.ncbi.nlm.nih.gov/sites/?cmd=HistorySearch&querykey=19&tab=&) |
| [#14](http://www.ncbi.nlm.nih.gov/sites/?querykey=19&dbase=pubmed&tab=History&querytype=eSearch&) | #7 OR #13 | [2831](http://www.ncbi.nlm.nih.gov/sites/?cmd=HistorySearch&querykey=19&tab=&) |
| [#13](http://www.ncbi.nlm.nih.gov/sites/?querykey=21&dbase=pubmed&tab=History&querytype=eSearch&) | #11 OR #12 | [862](http://www.ncbi.nlm.nih.gov/sites/?cmd=HistorySearch&querykey=21&tab=&) |
| [#12](http://www.ncbi.nlm.nih.gov/sites/?querykey=20&dbase=pubmed&tab=History&querytype=eSearch&) | #8 AND #9 | [756](http://www.ncbi.nlm.nih.gov/sites/?cmd=HistorySearch&querykey=20&tab=&) |
| [#11](http://www.ncbi.nlm.nih.gov/sites/?querykey=19&dbase=pubmed&tab=History&querytype=eSearch&) | #8 AND #10 | [139](http://www.ncbi.nlm.nih.gov/sites/?cmd=HistorySearch&querykey=21&tab=&) |
| [#10](http://www.ncbi.nlm.nih.gov/sites/?querykey=18&dbase=pubmed&tab=History&querytype=eSearch&) | hemorrhag? AND [humans]/lim | [177862](http://www.ncbi.nlm.nih.gov/sites/?cmd=HistorySearch&querykey=19&tab=&) |
| [#9](http://www.ncbi.nlm.nih.gov/sites/?querykey=21&dbase=pubmed&tab=History&querytype=eSearch&) | 'fever'/exp OR fever AND [humans]/lim | [140290](http://www.ncbi.nlm.nih.gov/sites/?cmd=HistorySearch&querykey=21&tab=&) |
| [#8](http://www.ncbi.nlm.nih.gov/sites/?querykey=20&dbase=pubmed&tab=History&querytype=eSearch&) | crimean OR 'congo'/exp OR congo | [9160](http://www.ncbi.nlm.nih.gov/sites/?cmd=HistorySearch&querykey=20&tab=&) |
| [#7](http://www.ncbi.nlm.nih.gov/sites/?querykey=19&dbase=pubmed&tab=History&querytype=eSearch&) | #5 AND #6 | [7282](http://www.ncbi.nlm.nih.gov/sites/?cmd=HistorySearch&querykey=19&tab=&) |
| [#6](http://www.ncbi.nlm.nih.gov/sites/?querykey=18&dbase=pubmed&tab=History&querytype=eSearch&) | 'tribavirin'/exp OR tribavirin OR 'virazole'/exp OR virazole OR 'ribavirin'/exp OR ribavirin AND [humans]/lim | [5498](http://www.ncbi.nlm.nih.gov/sites/?cmd=HistorySearch&querykey=18&tab=&) |
| [#5](http://www.ncbi.nlm.nih.gov/sites/?querykey=15&dbase=pubmed&tab=History&querytype=eSearch&) | **#1 or #2 or #3 or #4** | [2512](http://www.ncbi.nlm.nih.gov/sites/?cmd=HistorySearch&querykey=15&tab=&) |
| [#4](http://www.ncbi.nlm.nih.gov/sites/?querykey=14&dbase=pubmed&tab=History&querytype=eSearch&) | hemorrhagic AND fever, AND ('congo'/exp OR congo) AND [humans]/lim | [508](http://www.ncbi.nlm.nih.gov/sites/?cmd=HistorySearch&querykey=14&tab=&) |
| [#3](http://www.ncbi.nlm.nih.gov/sites/?querykey=13&dbase=pubmed&tab=History&querytype=eSearch&) | hemorrhagic AND ('fever'/exp OR fever) AND crimean AND [humans]/lim | [359](http://www.ncbi.nlm.nih.gov/sites/?cmd=HistorySearch&querykey=13&tab=&) |
| [#2](http://www.ncbi.nlm.nih.gov/sites/?querykey=10&dbase=pubmed&tab=History&querytype=eSearch&) | viral AND hemorrhagic AND ('fever' OR 'fever'/exp OR fever) AND [humans]/lim | [2266](http://www.ncbi.nlm.nih.gov/sites/?cmd=HistorySearch&querykey=10&tab=&) |
| [#1](http://www.ncbi.nlm.nih.gov/sites/?querykey=8&dbase=pubmed&tab=History&querytype=eSearch&) | crimean AND ('congo' OR 'congo'/exp OR congo) AND hemorrhagic AND ('fever' OR 'fever'/exp OR fever) AND [humans]/lim | [324](http://www.ncbi.nlm.nih.gov/sites/?cmd=HistorySearch&querykey=8&tab=&) |

ISI Web of Knowledge ([www.**isiknowledge**.com](http://www.isiknowledge.com) ): Search strategy performed on 25^th^ September 2009

| **#** | **Search strategy** | **Result** |
| --- | --- | --- |
| [#4](http://www.ncbi.nlm.nih.gov/sites/?querykey=14&dbase=pubmed&tab=History&querytype=eSearch&) | #3 OR #2 OR #1  *Timespan=All Years* | [106](http://www.ncbi.nlm.nih.gov/sites/?cmd=HistorySearch&querykey=14&tab=&) |
| [#3](http://www.ncbi.nlm.nih.gov/sites/?querykey=13&dbase=pubmed&tab=History&querytype=eSearch&) | Topic=(CONGO Hemorrhag* Fever) AND Topic=(tribavirin OR virazole OR ribavirin)  *Timespan=All Years* | [105](http://www.ncbi.nlm.nih.gov/sites/?cmd=HistorySearch&querykey=13&tab=&) |
| [#2](http://www.ncbi.nlm.nih.gov/sites/?querykey=10&dbase=pubmed&tab=History&querytype=eSearch&) | Topic=(Crimean Hemorrhag* Fever) AND Topic=(tribavirin OR virazole OR ribavirin)  *Timespan=All Years* | [96](http://www.ncbi.nlm.nih.gov/sites/?cmd=HistorySearch&querykey=10&tab=&) |
| [#1](http://www.ncbi.nlm.nih.gov/sites/?querykey=8&dbase=pubmed&tab=History&querytype=eSearch&) | Topic=(Crimean Congo Hemorrhag* Fever) AND Topic=(tribavirin OR virazole OR ribavirin)  *Timespan=All Years* | [95](http://www.ncbi.nlm.nih.gov/sites/?cmd=HistorySearch&querykey=8&tab=&) |

Google Scholar ([www.**scholar.google.co.uk**](http://www.scholar.google.co.uk) ): Search strategy performed on 26^th^ September 2009

| **#** | **Search strategy** | **Result** |
| --- | --- | --- |
| [#4](http://www.ncbi.nlm.nih.gov/sites/?querykey=14&dbase=pubmed&tab=History&querytype=eSearch&) | #3 and #2 and #1  *Timespan=All Years* | [566](http://www.ncbi.nlm.nih.gov/sites/?cmd=HistorySearch&querykey=14&tab=&) |
| [#3](http://www.ncbi.nlm.nih.gov/sites/?querykey=13&dbase=pubmed&tab=History&querytype=eSearch&) | Limitations:  “anywhere in the article”  “return only articles in the following subject areas: Medicine, pharmacology and veterinary science” |  |
| [#2](http://www.ncbi.nlm.nih.gov/sites/?querykey=10&dbase=pubmed&tab=History&querytype=eSearch&) | RIBAVIRIN (with the **exact phrase**) |  |
| [#1](http://www.ncbi.nlm.nih.gov/sites/?querykey=8&dbase=pubmed&tab=History&querytype=eSearch&) | crimean congo hemorrhagic fever (with **all** of the words) |  |

Cochrane Library: Search strategy performed on 26^th^ September 2009

| **#** | **Search strategy** | **Result** |
| --- | --- | --- |
| [#3](http://www.ncbi.nlm.nih.gov/sites/?querykey=13&dbase=pubmed&tab=History&querytype=eSearch&) | [(crimean congo):ti,ab,kw](http://www3.interscience.wiley.com/cochrane/searchHistory?mode=runquery&qnum=1) | [0](http://www.ncbi.nlm.nih.gov/sites/?cmd=HistorySearch&querykey=14&tab=&) |
| [#2](http://www.ncbi.nlm.nih.gov/sites/?querykey=10&dbase=pubmed&tab=History&querytype=eSearch&) | [MeSH descriptor **Hemorrhagic Fever Virus, Crimean-Congo** explode all trees](http://www3.interscience.wiley.com/cochrane/searchHistory?mode=runquery&qnum=2) | [0](http://www.ncbi.nlm.nih.gov/sites/?cmd=HistorySearch&querykey=14&tab=&) |
| [#1](http://www.ncbi.nlm.nih.gov/sites/?querykey=8&dbase=pubmed&tab=History&querytype=eSearch&) | crimean congo hemorrhagic fever | [0](http://www.ncbi.nlm.nih.gov/sites/?cmd=HistorySearch&querykey=14&tab=&) |

metaRegister of Controlled Trials (mRCT) – active register ([www.controlled-trials.com/mrct](http://www.controlled-trials.com/mrct)): Search strategy performed on 25^th^ September 2009

| **#** | **Search strategy** | **Result** |
| --- | --- | --- |
| [#1](http://www.ncbi.nlm.nih.gov/sites/?querykey=8&dbase=pubmed&tab=History&querytype=eSearch&) | Crimean congo hemorrhagic fever   - ISRCTN Register - trials registered with a unique identifier - National Health Service Research and Development Health Technology Assessment Programme (HTA) - Action Medical Research - National Institutes of Health (NIH) - randomized trial records held on NIH *ClinicalTrials.gov* website. - The Wellcome Trust Medical Research Council (UK) - UK Clinical Trials Gateway - Pilot | [0](http://www.ncbi.nlm.nih.gov/sites/?cmd=HistorySearch&querykey=14&tab=&) |
