# Supplementary material for: Ribavirin for Crimean-Congo hemorrhagic fever: systematic review and meta-analysis
Source: BMC Infect Dis. 2010 Jul 13;10:207. doi: 10.1186/1471-2334-10-207 (PMC2912908; doi:10.1186/1471-2334-10-207)
Supplement: Additional file 2 — Description of studies (table). [file 1471-2334-10-207-S2.DOCX]

Title: Characteristic of included studies

Description: this table describes all studies included in this review. It reports on the study type, the duration of follow up, the number of suspected and confirmed cases, mean age, gender and diagnostic criteria used; the dose of ribavirin, the days until onset of symptoms, any supportive therapy, and the mortality reported

| **Study Name** | **Design** | | **Population characteristics** | | | | **Intervention characteristics** | | | **Mortality per group** |
| --- | --- | --- | --- | --- | --- | --- | --- | --- | --- | --- |
|  | **Study Type** | **Follow-up duration** | **N suspected /confirmed cases** | **Age (mean/SD)** | **Gender (% female)** | **Criteria for confirmation of cases** | **Dose (loading, Initial and Maintenance)/ Route of administration** | **No. days until onset of symptoms (mean/SD)** | **Type of Supportive Therapy** |  |
| **Iran** | | | | | | | | | | |
| **Alavi-Naimi 2006 [1, 2]** | Historical control | NR | 255/155 | 32.05 (15.3) | 22% | PCR, IgM and IgG, antibodies, ELISA | 30mg/kg once; 15mg/kg every 5 hr for 4 days; 7.5 mg/kg 3x daily for 6 days | 4.4 (2.6) | NR | Ribavirin: 37/236  Untreated: 12/19 |
| **Izadi 2009 [3, 4]** | Case-series | NA | 179/63 | 29-37 (range: 11-75) | 19% | PCR, IgM and IgG antibodies, Virus antigens, ELISA | 30mg/kg once; 15mg/kg 4x daily for 4 days; 7.5 mg/kg 3x daily for 6 days | NR | Supportive therapy and blood products | Ribavirin (within 5 days): 16/109  Ribavirin (after 5 days): 18/70 |
| **Mardani 2003 [5, 6]** | Historical control | NA | 187/81 | 103 suspected cases < 33yrs; 84 ≥ 33 yrs | 31.5% | IgM and IgG antibodies, viral culture, ELISA | 30mg/kg once; 15mg/kg 4x daily for 4 days; 7.5 mg/kg 3x daily for 6 days | 4 | NR | Ribavirin (all cases): 42/139  Untreated (all cases): 22/48  Ribavirin (conf. cases): 8/69  Untreated (conf. cases): 7/12 |
| **Salehi 2004 [7]** | Single blinded (staff), randomized controlled clinical trial | One week | 60/40 | Range:21-50 | 35% | ELISA for specific IgM and IgG | 30 mg/kg once; 16 mg/kg every 6 hours for 4 days; 8 mg/kg every 8 hours for 6 days  12 patients received 30-50 mg/kg dose of IVIG | 3-8 days | Electrolytes, calorie, anti-fever agents, oxygen, suction of bronchial secretion | Intervention group (ribavirin + IVIG): 3/12 (confirmed cases);  Control group (ribavirin only): 3/28 (confirmed cases) |
| **Sharifi-Mood 2008 [8-10]** | Cross-sectional | NA | NR/34 | 13.3 (4.6) | 32% | PCR, IgM antibodies, genomic segments of CCHF, ELISA | 30mg/kg once; 15mg/kg every 4x daily for 4 days; 7.5 mg/kg 3x daily for 6 days | Median: 3 | NR | Early Ribavirin use: 9/34 |
| **Sharifi-Mood 2009 [11]** | Cross-sectional | NA | NR/123 | NR | NR | PCR, ELISA | NR | NR | NR | Early Ribavirin use: 2/91  Late Ribavirin use: 17/32 |
| **Pakistan** | | | | | | | | | | |
| **Jamil 2005 [12]** | Case series | 3 months | 8/6 | 26.4 (range: 15-48) | 0% | PCR, IgM and IgG antibodies, viral antigens, genomic segments of CCHF | 400mg once; 400mg 4x daily for 2 to 4 days | 4.3 | Supportive therapy and blood products | Ribavirin: 4/5  Untreated: 2/3  One patient in each group had not being confirmed as having CCHF |
| **Nadeem 2003 [13, 14]** | Historical Control | 5 months | 84/34 | 32 (range: 4-75) | 23% | PCR, IgM and IgG antibodies, viral antigens | NR | NR | Supportive therapy and blood products | Ribavirin: 4/34  Untreated: 4/4 |
| **Sheikh 2005 [15, 16]** | Case-series | Over 60 months | 135/83 | 33.5 (18.7) | 36% | PCR, IgM and IgG antibodies, viral culture, ELISA | 2g once; 1g 4x daily for 4 days; 0.5g 4x daily for 6 days | 6.2 (0.76) | Supportive therapy and blood products | Ribavirin: 8/83 |
| **Turkey** | | | | | | | | | | |
| **Ayten 2008 [17]** | Case-series | 24 months | 63/unclear | 46 (16.9) | 50.8% | PCR, IgM antibodies, genomic segments of CCHF, ELISA | NR | 4.7 (2.5) | Supportive therapy | Unclear but overall case fatality rate was 4.8% |
| **Bodur 2008 [18]** | Historical control | NR | NR/40 | 40-48 | 45% | PCR, genomic segments of the CCHF virus | 4g per day for 4 days; 2.4g per day for 6 days | 4.5-5 | Supportive therapy and blood products | Ribavirin: 1/8  Control: 5/32 |
| **Cevik 2008 [19, 20]** | Historical control | 4 months | NR/25 | NR | 44% | PCR, IgM antibodies, ELISA | 17mg/kg once; 17mg/kg 4x daily for 4 days; 8mg/kg 3x daily for 6 days | NR | Supportive therapy and blood products | Ribavirin: 5/9  Untreated: 7/16 |
| **Elaldi 2009 [21-23]** | Historical control | 12 months | 258/218 | 40.9-44.4 (16.7-19.1) | 50% | IgM and IgG antibodies, virus antigens | 30mg/kg once; 15mg/kg 4x daily for 4 days; 7.5 mg/kg 3x daily for 6 days | Median: 5 | Supportive therapy and blood products | Ribavirin: 9/126  Untreated: 11/92  Only data on confirmed patients is provided |
| **Ergonul 2006 [24, 25]** | Historical control | 36 months | NR/54 | 43-54 (11-18) | 52% | PCR, IgM antibodies, genomic segments of the CCHF virus, ELISA | 4g 4x daily for 4 days; 2.5g 4x daily for 6 days | NR | Supportive therapy and blood products | Ribavirin: 1/22  Untreated: 3/23 |
| **Ergonul 2007 [26]** | Cohort | 36 months | 75/1 | Median :30 | 68% | IgM and IgG antibodies, ELISA | NR | NR | NR | NR |
| **Ertugrul 2009 [27]** | Historical control | 13 months | 61/26 | 30.7 (20.6) | 42% | PCR, IgM and IgG antibodies, genomic segments of the CCHF virus, ELISA | NR | 3 | Supportive therapy and blood products | Ribavirin: 1/17  Untreated: 0/9 |
| **Fisgin 2009 [28-30]** | Historical control | 42 months | NR/53 | 44-51 (15-16) | 34.6% | PCR, IgM and IgG antibodies, ELISA | NR | NR | NR | Early Ribavirin use: 6/21  Late Ribavirin use: 7/21  Untreated: 5/11 |
| **Koksal 2009 [31, 32]** | Randomized trial | NR | NR/136 | 46.2-49.2 (17-18.5) | 32.4% | PCR, IgM and IgG antibodies, genomic segments of the CCHF virus, ELISA | 30mg/kg once; 15mg/kg 4x daily for 4 days; 7.5 mg/kg 3x daily for 6 days | Ribavirin Group: 4.5 (2.5); Supportive therapy Group: 3.9 (2.4) days | Supportive therapy and blood products | Ribavirin: 4/64  Untreated: 4/72 |
| **Midili 2007 [33]** | Cohort | 5 months | 91/10 | 40 (range: 13-67) | 60% | PCR, IgM and IgG antibodies, genomic segments of the CCHF virus | NR | Median: 6.5 (range: 3-7) | Supportive therapy and blood products | Ribavirin: 0/10 |
| **Ozkurt 2006 [34]** | Case-control | 24 months | 60/36  120 healthy controls taken from population | 40 (17) | 46.7% | IgM and IgG antibodies, genomic segments of the CCHF virus, ELISA | 2000mg once; 1000g 4x daily for 4 days; 500g 4x daily for 6 days | NR | Supportive therapy and blood products | Ribavirin: 2/22  Untreated: 4/38  Control: 13/120  Unclear how many patients in each group had not being confirmed as having CCHF |
| **Russia** | | | | | | | | | | |
| **Sannikova 2007 [35]** | Case series | NR | 283 confirmed cases | NR | NR | PCR and ELISA | 2000mg once; 1000-1200g daily for 2 days | NR | Supportive therapy and blood products | All patients received ribavirin, but mortality not reported |

**References of included studies**

1. Alavi-Naini R, Moghtaderi A, Koohpayeh HR, Sharifi-Mood B, Naderi M, Metanat M, Izadi M: **Crimean-Congo hemorrhagic fever in Southeast of Iran.** *Journal of Infection* 2006, **52:**378-382.

2. Mardani M, Goya M, Zainali M, Jahromi MK: **Clinico-epidemiologic feature and outcome analysis of Crimean-Congo haemorraghic fever in Iran (1999-2006).** *International Journal of Antimicrobial Agents* 2007, **29:**S280-S280.

3. Izadi S, Salehi M: **Evaluation of the efficacy of ribavirin therapy on survival of Crimean-Congo hemorrhagic fever patients: A case-control study.** *Japanese Journal of Infectious Diseases* 2009, **62:**11-15.

4. Metanat M, Sharifi-Mood B, Salehi M: **A Comparative Study of the Efficacy of Oral Ribavirin for Crimean-Congo Hemorrhagic Fever in Patients Treated during the Initial 5 Days vs after 5 Days of the Disease, Southeast Iran.** *International Journal of Antimicrobial Agents* 2005, **26:**S85-S85.

5. Mardani M, Jahromi MK, Naieni KH, Zeinali M: **The efficacy of oral ribavirin in the treatment of Crimean-Congo hemorrhagic fever in Iran.** *Clinical Infectious Diseases* 2003, **36:**1613-1618.

6. Mardani M, Keshtkar J, Holakoi N, Zinali M: **The efficacy of oral ribavirin in the treatment of 81 proved cases of Crimean-Congo hemorrhagic fever in Iran (1991-2001).** *Med J Islamic Republic of Iran* 2003, **3:**193-195.

7. Salehi H: **A comparative study on the effect of ribaverin with and without IVIG (Intravenous imuneglobuliu) in treatment of Crimean-congo hemorrhagic Fever (CCHF).** *Journal of Mazandaran University of Medical Sciences* 2004, **14:**33-38.

8. Sharifi-Mood B, Alavi-Naini R, Metanat M, Rakhshani F: **Ribavirin: An Effective Drug for Treatment of Children with Crimean-Congo Hemorrhagic Fever: A Seven-years Experience.** *Pakistan Journal of Biological Sciences* 2006, **9:**1598-1600.

9. Sharifi-Mood B, Mardani M, Hatami H, Metanat M: **Crimean-Congo haemorrhagic fever among children in Southeast Iran (clinico-epidemiological feature and outcome analysis).** *International Journal of Antimicrobial Agents* 2007, **29:**S48-S49.

10. Sharifi-Mood B, Mardani M, Keshtkar-Jahromi M, Rahnavardi M, Hatami H, Metanat M: **Clinical and epidemiologic features of Crimean-Congo hemorrhagic fever among children and adolescents from southeastern Iran.** *Pediatric Infectious Disease Journal* 2008, **27:**561-563.

11. Sharifi-Mood B, Metanat M, Ghorbani-Vaghei A, Fayyaz-Jahani F, Akrami E: **The outcome of patients with Crimean-Congo hemorrhagic fever in Zahedan, southeast of Iran: a comparative study.** *Archives of Iranian medicine* 2009, **12:**151-153.

12. Jamil B, Hasan RS, Sarwari AR, Burton J, Hewson R, Clegg C: **Crimean-Congo hemorrhagic fever: Experience at a tertiary care hospital in Karachi, Pakistan.** *Transactions of the Royal Society of Tropical Medicine and Hygiene* 2005, **99:**577-584.

13. Ali N, Chotani R, Anwar M, Nadeem M, Karamat K, Tariq W: **A crimean-congo haemorrhagic Fever outbreak in northern balochistan.** *Journal of the College of Physicians and Surgeons--Pakistan: JCPSP* 2007, **17:**477.

14. Nadeem M, Ali N, Anwar M, Hussain I, Mohammad T, Hayee A: **A comparision of clinical diagnosis and serological diagnosis in an epidemic of Crimean-Congo Haemorrhagic Fever.** *Pakistan Journal of Medical Sciences* 2003, **19:**247-251.

15. Sheikh AS, Sheikh AA, Sheikh NS, Rafi US, Asif M, Afridi F, Malik MT: **Bi-annual surge of Crimean-Congo haemorrhagic fever (CCHF): A five-year experience.** *International Journal of Infectious Diseases* 2005, **9:**37-42.

16. Sheikh AS, Sheikh AA, Sheikh NS, Tariq M: **Ribavirin: An effective treatment of Crimean-Congo Haemorrhagic Fever.** *Pakistan Journal of Medical Sciences* 2004, **20:**201-206.

17. Ayten K, Kemalettin O, Serpil E, Mehmet P: **Crimean-Congo haemorrhagic fever virus infection in eastern Turkey.** In *The second International Congress of Central Asia Infectious Diseases (ICCAID); Almaty, Kazakhstan*. [www.iccaid.org;](http://www.iccaid.org;) 2008

18. Bodur H, Erbay A, Akinci E, Onguru P, Bayazit N, Eren S, Kubar A: **Effect of Oral Ribavirin Treatment to the Viral Load and Progress of the Disease in Crimean Congo Hemorrhagic Fever.** *Abstracts of the Interscience Conference on Antimicrobial Agents and Chemotherapy* 2008, **48:**713-714.

19. Cevik M, Elaldi N, AkiNci E, Oenguerue P, Erbay A, Buzgan T, Uzun R, Kubar A, Boduri H: **A Preliminary Study to Evaluate the Effect of Intravenous Ribavirin Treatment on Survival Rates in Crimean-Congo Hemorrhagic Fever.** *Abstracts of the Interscience Conference on Antimicrobial Agents and Chemotherapy* 2007, **47:**487.

20. Cevik MA, Elaldi N, Akinci E, Onguru P, Erbay A, Buzgan T, Uzun R, Kubar A, Bodur H: **A preliminary study to evaluate the effect of intravenous ribavirin treatment on survival rates in Crimean-Congo hemorrhagic fever.** *Journal of Infection* 2008, **57:**350-351.

21. Elaldi N, Bodur H, Ascioglu S, Celikbas A, Ozkurt Z, Vahaboglu H, Leblebicioglu H, Yilmaz N, Engin A, Sencan M, et al: **Efficacy of oral ribavirin treatment in Crimean-Congo haemorrhagic fever: A quasi-experimental study from Turkey.** *Journal of Infection* 2009, **58:**238-244.

22. Elaldi N, Bodur H, Celikbas A, Ozkurt Z, Leblebicioglu H, Bakir M, Aydin K, Yilmaz N, Dokmetas I, Cevik MA, et al: **Comparison of oral ribavirin treatment in Crimean-Congo haemorrhagic fever: a historical cohort study in Turkey.** *International Journal of Antimicrobial Agents* 2007, **29:**S48-S48.

23. Elaldi N, Kaya S, Gursoy N, Gunes T, Engin A, Kubar A, Karakus G, Polat ZA, Yilmaz M, Yilmaz N, et al: **Efficacy of Oral Ribavirin Treatment on Serum Viral Loads and Cytokine Levels in Severe Crimean-Congo Hemorrhagic Fever (CCHF) Cases.** *Abstracts of the Interscience Conference on Antimicrobial Agents and Chemotherapy* 2007, **47:**488.

24. Ergonul O, Celikbas A, Baykam N, Eren S, Dokuzoguz B: **Analysis of risk-factors among patients with Crimean-Congo haemorrhagic fever virus infection: severity criteria revisited.** *Clin Microbiol Infect* 2006, **12:**551-554.

25. Ergonul O, Celikbas A, Dokuzoguz B, Eren S, Baykam N, Esener H: **Characteristics of patients with Crimean-Congo hemorrhagic fever in a recent outbreak in Turkey and impact of oral ribavirin therapy.** *Clin Infect Dis* 2004, **39:**284-287.

26. Ergonul O, Zeller H, Celikbas A, Dokuzoguz B: **The lack of Crimean-Congo hemorrhagic fever virus antibodies in healthcare workers in an endemic region.** *International Journal of Infectious Diseases* 2007, **11:**48-51.

27. Ertugrul B, Uyar Y, Yavas K, Turan C, Oncu S, Saylak O, Carhan A, Ozturk B, Erol N, Sakarya S: **An outbreak of Crimean-Congo hemorrhagic fever in western Anatolia, Turkey.** *International Journal of Infectious Diseases* 2009.

28. Fisgin NT, Doganci L, Ergonul O, Tulek N: **Early Ribavirin Use in Crimean-Congo Hemorrhagic Fever: Significant Positive Impact in Prognosis.** *Abstracts of the Interscience Conference on Antimicrobial Agents and Chemotherapy* 2008, **48:**714.

29. Fisgin NT, Ergonul O, Doganci L, Tulek N: **The role of ribavirin in the therapy of crimean-congo hemorrhagic fever: Early use is promising.** *European Journal of Clinical Microbiology and Infectious Diseases* 2009, **28:**929-933.

30. Fisgin NT, Tanyel E, Doganci L, Tulek N: **Risk factors for fatality in patients with Crimean-Congo haemorrhagic fever.** *Tropical Doctor* 2009, **39:**158-160.

31. Koksal I, Yilmaz G, Aksoy F, Aydin H, Yavuz I, Iskender S, Akcay K, Erensoy S, Aydin K: **The Efficacy of Ribavirin in the Treatment of Crimean-Congo Hemorrhagic Fever.** *Abstracts of the Interscience Conference on Antimicrobial Agents and Chemotherapy* 2008, **48:**717.

32. Koksal I, Yilmaz G, Aksoy F, Aydin H, Yavuz I, Iskender S, Akcay K, Erensoy S, Caylan R, Aydin K: **The efficacy of ribavirin in the treatment of Crimean-Congo hemorrhagic fever in Eastern Black Sea region in Turkey.** *Journal of Clinical Virology* 2009, **In Press, Corrected Proof**.

33. Midilli K, Gargili A, Ergonul O, Sengoz G, Ozturk R, Bakar M, Jongejan F: **Imported Crimean-Congo hemorrhagic fever cases in Istanbul.** *BMC infectious diseases* 2007, **7:**54.

34. Ozkurt Z, Kiki H, Erol S, Erdem F, Yilmaz N, Partak M, Gundogdu M, Tasyaran MA: **Crimean-Congo hemorrhagic fever in Eastern Turkey: clinical features, risk factors and efficacy of ribavirin therapy.** *Journal of Infection* 2006, **52:**207-215.

35. Sannikova IV, Kliushnikov Iu I, Popov PN, Sysoliatina GV, Evchenko Iu M, Shenetts KV, Popov VA, Marchukova LN: **[Clinico-epidemiological characterization of Crimean haemorrhagic fever in Stavropol' region].** *Zh Mikrobiol Epidemiol Immunobiol* 2001**:**89-92.
