# Supplementary material for: Ribavirin for Crimean-Congo hemorrhagic fever: systematic review and meta-analysis
Source: BMC Infect Dis. 2010 Jul 13;10:207. doi: 10.1186/1471-2334-10-207 (PMC2912908; doi:10.1186/1471-2334-10-207)
Supplement: Additional file 3 — Risk of bias of included studies (table). [file 1471-2334-10-207-S3.DOCX]

Title. Risk of bias of included studies

Description: An assessment of the risk of bias assessed by Downs & Black checklist of all included studies

## Reporting

| **Study** | **Country** | **Study design** | **Q1: Aim clearly described** | **Q2: Outcomes clearly described** | **Q3: Patients chracteristics clearly described** | **Q4: Interventions clearly described** | **Q5: Principal confounders clearly described** | **Q6: Main findings clearly described** | **Q7: Random variability for the main outcome provided** | **Q8: Adverse events reported** | **Q9: Lost to follow up reported** | **Q10: Actual p-value reported** |
| --- | --- | --- | --- | --- | --- | --- | --- | --- | --- | --- | --- | --- |
| Alavi-Naimi 2006 | Iran | Historical control | Yes | No | Yes | No | Partially | Yes | Yes | No | No | Yes |
| Ayten 2008 | Turkey | Case-series | No | No | No | No | No | No | No | No | No | No |
| Bodur 2008 | Turkey | Historical control | Yes | No | No | No | No | No | No | No | No | Yes |
| Cevik 2008 | Turkey | Historical control | Yes | No | No | Yes | No | No | Yes | Yes | No | Yes |
| Elaldi 2009 | Turkey | Historical control | Yes | Yes | Yes | Yes | Partially | Yes | Yes | No | No | Yes |
| Ergonul 2006 | Turkey | Cohort | Yes | No | No | No | No | No | No | Yes | No | Yes |
| Ergonul 2007 | Turkey | Cohort | Yes | No | No | No | Yes | No | No | No | No | No |
| Ertugrul 2009 | Turkey | Cross-sectional | Yes | No | No | No | No | No | Yes | No | No | No |
| Fisgin 2009 | Turkey | Historical control | Yes | No | No | No | No | No | Yes | No | No | No |
| Izadi 2009 | Iran | Case-series | Yes | No | Yes | No | Partially | No | Yes | No | No | No |
| Jamil 2005 | Pakistan | Case-series | Yes | No | Yes | No | No | No | No | No | No | No |
| Koksal 2008 | Turkey | RCT | Yes | Yes | Yes | Yes | Yes | Yes | Yes | Yes | No | Yes |
| Mardani 2003 | Iran | Historical control | Yes | No | No | No | Partially | Yes | Yes | No | No | No |
| Midilli 2007 | Turkey | Cohort | Yes | Yes | Yes | No | Partially | No | Yes | No | No | No |
| Nadeem 2003 | Pakistan | Cohort | No | No | No | Yes | No | No | No | No | No | No |
| Ozkurt 2006 | Turkey | Historical control | Yes | Yes | No | No | Partially | No | Yes | Yes | Yes | Yes |
| Salehi 2004 | Iran | RCT | Yes | Yes | No | Yes | No | Yes | No | No | No | No |
| Sannikova 2007 |  | Case-series | Yes | No | No | Yes | No | Yes | No | No | No | No |
| Sharifi-Mood 2008 | Iran | Cross-sectional | No | No | No | Yes | Partially | No | No | No | No | Yes |
| Sharifi-Mood 2009 | Iran | Cross-sectional | No | No | No | No | Partially | No | No | No | No | No |
| Sheikh 2005 | Pakistan | Case-series | No | No | No | Yes | No | No | No | No | No | No |

## External validity and Bias

| **Study** | **Country** | **Study design** | **Q11: Sample asked to participate representative of the population** | **Q12: Sample agreed to participate representative of the population** | **Q13: Staff participating representative of the patient's environment** | **Q14: Attempt to blind participants** | **Q15: Attempt to blind assessors** | **Q16: Data dredging results stated clearly** | **Q17: Analysis adjusted for length of follow up** | **Q18: Appropriate statistics** | **Q19: Reliable compliance** | **Q20: Accurate outcome measures** |
| --- | --- | --- | --- | --- | --- | --- | --- | --- | --- | --- | --- | --- |
| Alavi-Naimi 2006 | Iran | Historical control | Yes | Yes | Unable to determine | Unable to determine | Unable to determine | Unable to determine | Unable to determine | Yes | Unable to determine | Yes |
| Ayten 2008 | Turkey | Case-series | Unable to determine | Unable to determine | Unable to determine | No | No | Unable to determine | No | Unable to determine | Unable to determine | Unable to determine |
| Bodur 2008 | Turkey | Historical control | Unable to determine | Unable to determine | Unable to determine | No | No | Unable to determine | Unable to determine | Unable to determine | Unable to determine | Unable to determine |
| Cevik 2008 | Turkey | Historical control | Unable to determine | Unable to determine | Unable to determine | No | No | Yes | Yes | Yes | Unable to determine | Unable to determine |
| Elaldi 2009 | Turkey | Historical control | Yes | Unable to determine | Unable to determine | No | Unable to determine | Unable to determine | Yes | Yes | Unable to determine | Yes |
| Ergonul 2006 | Turkey | Cohort | Unable to determine | Unable to determine | Unable to determine | No | No | Unable to determine | No | Unable to determine | Unable to determine | Unable to determine |
| Ergonul 2007 | Turkey | Cohort | Unable to determine | Unable to determine | Unable to determine | No | No | Unable to determine | No | Unable to determine | Unable to determine | Unable to determine |
| Ertugrul 2009 | Turkey | Cross-sectional | Unable to determine | Unable to determine | Unable to determine | No | No | Unable to determine | No | Unable to determine | Unable to determine | Unable to determine |
| Fisgin 2009 | Turkey | Historical control | Unable to determine | Unable to determine | Unable to determine | Unable to determine | Unable to determine | Unable to determine | No | Yes | Unable to determine | Unable to determine |
| Izadi 2009 | Iran | Case-series | Unable to determine | Unable to determine | Unable to determine | Unable to determine | Unable to determine | No | Yes | Yes | Unable to determine | Yes |
| Jamil 2005 | Pakistan | Case-series | Unable to determine | Unable to determine | Unable to determine | No | No | Unable to determine | Unable to determine | No | Unable to determine | No |
| Koksal 2008 | Turkey | RCT | Unable to determine | Unable to determine | Unable to determine | No | No | Yes | Unable to determine | Yes | Yes | Yes |
| Mardani 2003 | Iran | Historical control | Yes | Yes | Unable to determine | Unable to determine | Unable to determine | Yes | Yes | Yes | Unable to determine | Yes |
| Midilli 2007 | Turkey | Cohort | Unable to determine | Unable to determine | Unable to determine | No | No | Unable to determine | No | Unable to determine | Unable to determine | Unable to determine |
| Nadeem 2003 | Pakistan | Cohort | Unable to determine | Unable to determine | Unable to determine | No | No | Unable to determine | No | No | Unable to determine | No |
| Ozkurt 2006 | Turkey | Historical control | Unable to determine | Unable to determine | Unable to determine | No | No | Unable to determine | No | Yes | Unable to determine | Yes |
| Salehi 2004 | Iran | RCT | Yes | Yes | Yes | No | Yes | Unable to determine | Unable to determine | Unable to determine | Unable to determine | Yes |
| Sannikova 2007 | Russia | Case-series | Yes | Yes | Yes | No | No | Unable to determine | No | No | No | Unable to determine |
| Sharifi-Mood 2008 | Iran | Cross-sectional | Unable to determine | Unable to determine | Unable to determine | Unable to determine | Unable to determine | Unable to determine | No | Yes | Unable to determine | Yes |
| Sharifi-Mood 2009 | Iran | Cross-sectional | Unable to determine | Unable to determine | Unable to determine | Unable to determine | Unable to determine | Unable to determine | No | No | Unable to determine | Unable to determine |
| Sheikh 2005 | Pakistan | Case-series | Yes | Unable to determine | Unable to determine | No | No | Unable to determine | No | Unable to determine | Unable to determine | Unable to determine |

## Selection bias and power

| **Study** | **Country** | **Study design** | **Q21: Same population** | **Q22: Participants recruited at the same time** | **Q23: Randomised?** | **Q24: Adequate allocation concealment?** | **Q25: Adequate adjustment for confounders?** | **Q26: Loss of follow up reported?** | **Q27: Power calculation** |
| --- | --- | --- | --- | --- | --- | --- | --- | --- | --- |
| Alavi-Naimi 2006 | Iran | Historical control | Yes | yes | no | no | Unable to determine | Unable to determine | <n1 |
| Ayten 2008 | Turkey | Case-series | Unable to determine | Unable to determine | no | no | no | Unable to determine | <n1 |
| Bodur 2008 | Turkey | Historical control | Unable to determine | Unable to determine | no | Unable to determine | no | Unable to determine | <n1 |
| Cevik 2008 | Turkey | Historical control | Unable to determine | Unable to determine | no | no | no | Unable to determine | <n1 |
| Elaldi 2009 | Turkey | Historical control | Unable to determine | no | no | no | yes | no | <n1 |
| Ergonul 2006 | Turkey | Cohort | Unable to determine | Unable to determine | no | no | no | Unable to determine | <n1 |
| Ergonul 2007 | Turkey | Cohort | Unable to determine | Unable to determine | no | no | no | Unable to determine | <n1 |
| Ertugrul 2009 | Turkey | Cross-sectional | Unable to determine | Unable to determine | no | no | no | Unable to determine | <n1 |
| Fisgin 2009 | Turkey | Historical control | Unable to determine | Unable to determine | no | Unable to determine | no | Unable to determine | <n1 |
| Izadi 2009 | Iran | Case-series | Yes | yes | no | no | Unable to determine | Unable to determine | <n1 |
| Jamil 2005 | Pakistan | Case-series | Unable to determine | Unable to determine | no | no | no | no | <n1 |
| Koksal 2008 | Turkey | RCT | Yes | yes | Yes | Unable to determine | no | Unable to determine | <n1 |
| Mardani 2003 | Iran | Historical control | Yes | yes | no | no | Unable to determine | Unable to determine | <n1 |
| Midilli 2007 | Turkey | Cohort | Unable to determine | Unable to determine | no | no | no | Unable to determine | <n1 |
| Nadeem 2003 | Pakistan | Cohort | Unable to determine | Unable to determine | no | no | no | Unable to determine | <n1 |
| Ozkurt 2006 | Turkey | Historical control | Unable to determine | Unable to determine | no | no | no | Unable to determine | <n1 |
| Salehi 2004 | Iran | RCT | Yes | Yes | Unable to determine | Unable to determine | Unable to determine | Unable to determine | <n1 |
| Sannikova 2007 | Russia | Case-series | No | No | no | no | no | no | <n1 |
| Sharifi-Mood 2008 | Iran | Cross-sectional | Yes | yes | no | Unable to determine | Unable to determine | Unable to determine | <n1 |
| Sharifi-Mood 2009 | Iran | Cross-sectional | Yes | yes | no | Unable to determine | Unable to determine | Unable to determine | <n1 |
| Sheikh 2005 | Pakistan | Case-series | Unable to determine | Unable to determine | no | no | no | Unable to determine | <n1 |
